# A psychological network analysis of self-reported spatial cognitive strategies in commercial airline pilots and non-pilots

**DOI:** 10.3389/fnhum.2026.1844920

**Published:** 2026-08-17

**Authors:** Yuan Zhou, Ying Long, Shuqi Yao, Jing Zhang, Yuxuan Chen, Ruida Wang, Ming Chang

**Affiliations:** 1 School of Psychology, Shaanxi Normal University, Xi’an, China; 2 Shaanxi Provincial Key Laboratory of Behavior and Cognitive Neuroscience, Xi’an, China; 3 School of Physical Education, Shaanxi Normal University, Xi’an, China; 4 College of Sociology, Nankai University, Tianjin, China

**Keywords:** network analysis, pilot selection, spatial anxiety, spatial navigation, wayfinding strategy

## Abstract

**Objectives:**

Spatial navigation is a critical skill in aviation. Traditional research focusing on isolated scores cannot reveal the interactive architecture of cognitive components. This study employed psychological network analysis to compare the self-reported spatial cognitive strategy structures of airline pilots and non-pilot controls.

**Methods:**

In a cross-sectional design, 110 commercial airline pilots and 203 university students (non-pilot controls) completed self-report measures of spatial anxiety, wayfinding strategies, and sense of direction. Gaussian Graphical Models were used to estimate psychological networks for each group, followed by statistical comparison of their network structures.

**Results:**

The pilot group reported greater use of orientation strategies and higher self-rated navigation ability. The global network structure differed significantly between the two groups despite comparable overall connectivity. However, no individual edge or centrality index reached statistical significance after correction, suggesting a distributed rather than hub-driven reconfiguration. All findings were derived from self-report measures and reflect subjective strategy patterns rather than objective cognitive performance.

**Conclusion:**

The self-reported spatial strategy networks differed globally between the two groups, although no single edge or node drove the observed effect. These findings may reflect training effects, self-selection, or a combination of both, and the pronounced differences in sex and age distributions between the two groups may also have contributed to the observed network patterns. All inferences are limited to subjective strategy patterns rather than objective cognitive performance.

## 1 Introduction

Human spatial navigation constitutes a fundamental adaptive skill that underpins environmental exploration, migration, and the expansion of civilizations (Ekstrom and Isham, 2017; Kessler et al., 2024). In professions that place exceptionally high demands on spatial cognition, this fundamental cognitive capacity is honed into a specialized form of domain-specific expertise, where it directly influences operational efficiency and safety. Commercial aviation serves as a salient example. Pilots must continuously integrate multidimensional information within complex, dynamic, three-dimensional spaces, constructing and updating precise cognitive maps in real time to sustain a level of navigational awareness and situational perception characterized by a distinct spatial cognitive strategy profile differing from that of non-trained populations (Sutton et al., 2014; Tang et al., 2024). Consequently, investigating the cognitive architecture and underlying mechanisms of individuals who demonstrate specialized navigational proficiency, often termed spatial cognitive experts, has emerged as a central scientific inquiry. Pilot populations are frequently regarded as representative cases of such specialized expertise. Empirical research indicates that, compared to the general population, pilots tend to exhibit systematic differences on specific spatial-cognitive tasks. For example, student pilots receiving general aviation training were able to estimate directions more accurately after freely exploring a virtual town, a finding that suggests the formation of more precise cognitive maps (Sutton et al., 2014). Military pilots also demonstrate stronger performance on classic tests of small-scale spatial abilities, such as mental rotation and spatial metric relation judgment (Johnson et al., 2017). Furthermore, pilots score significantly higher than the general population on assessments of field independence, a cognitive style closely associated with spatial orientation, as evidenced in studies of Israeli Air Force personnel (Glicksohn and Naor-Ziv, 2016).

Crucially, these advantages are reflected not only in objective task performance but also in subjective self-assessment. Studies have found that pilot groups score significantly higher than non-pilot controls on the self-report Santa Barbara Sense of Direction Scale (SBSOD), a measure of environmental spatial ability. Moreover, their self-rated scores show significant correlations with performance on certain objective spatial tasks, such as object perspective-taking, which indicates that pilots possess more accurate self-assessments, or metacognitive accuracy, regarding their spatial abilities (Sutton et al., 2014). Collectively, this evidence delineates a characteristic profile of pilots as individuals with specialized spatial expertise: through rigorous selection and specialized training, they demonstrate a distinct pattern of strengths in constructing cognitive representations, processing spatial relations, and self-monitoring spatial abilities. Understanding the nature of this specialized advantage is therefore of fundamental importance for revealing the limits and plasticity of human spatial cognition, as well as for optimizing personnel selection and training protocols in occupations that impose substantial spatial processing demands (Hetmański, 2018).

Stable preferences in wayfinding strategies have been observed among individuals, ranging from a reliance on egocentric route information to the adoption of allocentric survey representations (Ekstrom et al., 2014). The formation of an accurate cognitive map, characterized by an allocentric and overhead spatial representation, has often been associated with navigational expertise (Ishikawa, 2023). Investigations into cognitive map construction specifically addressed these individual differences, illuminating why some individuals were found to integrate discrete routes into a unified map representation while others were not (Wolbers and Hegarty, 2010; Weisberg and Newcombe, 2015). Further evidence suggested that spatial knowledge acquired through route learning and cartographic map learning was dissociable, and that a preference for allocentric reference frames involved distinct neural substrates (Zhang et al., 2014). In line with this, the use of self-reported survey strategies was found to be associated with real-world environmental navigation performance, supporting the use of such self-reports as indicators of internal spatial representations (Ham et al., 2020). However, although individual strategy preferences could be captured at the item level by instruments such as the Wayfinding Strategy Scale (Lawton and Kallai, 2002; Bock et al., 2024), nuanced interactions between specific items assessing the tendency to construct mental maps and other cognitive-affective variables might have been obscured by traditional analytical approaches that aggregated item scores into composite indices or relied on mean comparisons (Fioriti et al., 2024), suggesting a potential need for a framework capable of modeling conditional dependencies among these elements. While the traditional “isolated-scores” paradigm has robustly documented pilots’ superior performance across discrete measures of spatial cognition, it is fundamentally constrained by its conceptualization of core constructs, such as spatial anxiety, wayfinding strategy, and self-assessed navigational efficacy, as independent or only linearly related variables (Glicksohn and Naor-Ziv, 2016; Oliver et al., 2023; Gentle et al., 2024). Consequently, although effective in identifying the specific dimensions on which experts excel, this approach cannot reveal the dynamic, reciprocal interactions among these cognitive and affective components that give rise to a coherent and efficient expert system.

As a complementary approach, psychological network analysis can reveal the dependency patterns among items rather than performing conventional dimension comparisons (Epskamp and Fried, 2018; Hevey, 2018). This theoretical perspective proposes that complex psychological constructs, including spatial cognition, emerge from dynamic networks of directly observable variables linked by reciprocal causal influences such as mutual activation, inhibition, or feedback (Orona et al., 2025). The methodology operationalizes this by treating measured variables as nodes and their conditional dependencies, estimated via indices like partial correlations, as edges. This enables both the visualization and quantitative characterization of a cognitive system’s architecture by modeling how components interact within the network structure (Epskamp and Fried, 2018). This approach has been successfully applied to identify more efficient and integrated network structures underlying expertise in related domains, including intelligence and motor skills, pinpointing core hubs and functional modules within those systems (Malanchini et al., 2020; Vagnetti et al., 2025). These demonstrations provide a strong methodological foundation for extending network analysis to spatial cognitive expertise. Nevertheless, this powerful analytical tool has not yet been employed to directly compare the self-reported spatial cognitive networks of real-world spatial experts, such as commercial airline pilots, with those of novice populations. This omission constitutes a critical empirical gap, leaving the specific connectivity patterns and systemic architecture that characterize expert spatial cognition largely unexamined. This gap consequently limits a deeper, system-level understanding of expert performance and hinders the translation of such insights into applied domains such as pilot selection and training.

Building upon the preceding theoretical perspective, the present study employed psychological network analysis to compare the network architectures derived from self-reported spatial anxiety, wayfinding strategies, and sense of direction between commercial airline pilots and a non-pilot control group. This analytical approach may help uncover inter-item dependency patterns that conventional dimension score comparisons tend to miss, thereby offering a novel perspective for understanding the cognitive organizational characteristics of expertise.

## 2 Methods

### 2.1 Study design and participants

This study comprised two distinct participant groups: a non-pilot control group and commercial airline pilots. Each group was recruited using a different sampling strategy. The non-pilot control group (*n* = 204) were recruited via convenience sampling, with the online survey link disseminated through university-related online forums and social media platforms. Commercial airline pilots (*n* = 111) were recruited using cluster sampling in collaboration with a major Chinese airline, with invitations extended during their scheduled recurrent training sessions. Participation was voluntary for all individuals.

Data were collected exclusively via an online survey platform. Participants first completed a demographic questionnaire. Subsequently, all participants completed three standardized self-report measures: the Spatial Anxiety Scale, the Wayfinding Strategy Inventory, and the Santa Barbara Sense of Direction Scale (SBSOD). The estimated completion time was between 15 and 20 min. Demographic characteristics for both groups are presented in Table 1.

**Table 1:** Demographic and sample characteristics of the participants.

Characteristic	Category/Description	Non-pilot control group (*n* = 203)	Commercial airline pilot (*n* = 110)
Gender, *n* (%)	Male	67 (33.0%)	109 (99.1%)
	Female	136 (67.0%)	1 (0.09%)
Age (years)	Mean (SD)	22.88 (2.74)	33.57 (7.97)
Key background	Undergraduate	64 (31.53%)	105 (95.45%)
	Dominant hand (right)	194 (95.6%)	89 (80.9%)

A total of 315 questionnaires were distributed. Following data collection, responses were screened based on criteria including excessively short completion time, patterned responding (straight-lining), or missing key data. This process yielded 313 valid questionnaires, resulting in a valid response rate of 99.4%. The final analytical sample consisted of *N* = 313 participants (203 in the non-pilot control group; 110 commercial airline pilots). Prior methodological research suggests that a sample size between 250 and 350 cases provides stable output for cross-sectional network analysis, characterized by high specificity, moderate sensitivity, and high correlation of edge weights (Epskamp et al., 2018; Burger et al., 2023). Our total sample size falls within this recommended range, thereby potentially supporting stable network estimation.

This study involved human participants and was approved by the Ethics Committee of Shaanxi Normal University (Approval No.: 202516056). All participants provided written informed consent prior to participation.

### 2.2 Measure

#### 2.2.1 Santa Barbara Sense of Direction Scale

SBSOD is a standardized self-report instrument designed to assess environmental spatial ability (Hegarty et al., 2002). It provides a rapid and psychometrically sound measure of self-assessed spatial navigation proficiency and has been widely validated in spatial cognition research. The Chinese version employed in this study was linguistically and culturally adapted. This study used the total score of the SBSOD as the indicator of self-reported spatial navigation ability. This adapted scale comprises 12 items measuring the unidimensional construct of sense of direction. Responses were recorded on a 7-point Likert scale ranging from 1 (strongly disagree) to 7 (strongly agree), with a midpoint of 4 (neutral). All items were positively scored, with higher scores indicating a stronger sense of direction; specifically, items 5, 6, 8, 9, 10, and 12 were reverse-scored prior to summing. A total score was computed by summing all items, with higher scores indicating a better self-reported sense of direction. In the current sample, the scale demonstrated good internal consistency, with a Cronbach’s alpha of 0.873.

#### 2.2.2 Spatial Anxiety Scale

The Spatial Anxiety Scale, originally developed by Lawton and Kallai (2002), was used to measure trait anxiety associated with navigation. The Chinese adapted version consists of 8 items, each describing a specific wayfinding scenario presumed to elicit anxiety (e.g., “finding your way to an appointment in an unfamiliar area of a city”). Participants rated their anticipated anxiety for each scenario on a 5-point Likert scale from 1 (not at all anxious) to 5 (very much anxious). A total score was derived by summing responses across all items, with higher scores reflecting greater spatial anxiety. For the non-pilot control group, the item “locating your car in a very large parking lot or parking garage” was excluded from analysis due to a low item-total correlation. The remaining 7-item version showed excellent internal consistency, with a Cronbach’s alpha of 0.896 in this study.

#### 2.2.3 Wayfinding Strategy Scale

Individual differences in large-scale spatial navigation were assessed using the Wayfinding Strategy Scale, which measures the propensity to employ two distinct cognitive strategies: route and orientation strategies. The Chinese version, adapted from Lawton’s original work, was administered (Lawton and Kallai, 2002). A route strategy is characterized by a primary reliance on landmarks and sequential turn-by-turn instructions. Conversely, an orientation strategy involves the use of cardinal directions, Euclidean distances, and configurational understanding of the environment to maintain a sense of direction.

The scale contains 9 items describing methods for locating a target in an urban or complex environment. Participants indicated how characteristic each method was of their typical behavior using a 5-point Likert scale (1 = not at all typical of me to 5 = extremely typical of me). The items form two subscales: a 5-item Orientation Strategy subscale and a 4-item Route Strategy subscale. Both subscales exhibited good reliability in the present study, with Cronbach’s alpha coefficients of 0.835 (Orientation) and 0.828 (Route).

### 2.3 Statistical analysis

The data analysis included descriptive statistics, correlation analysis, linear regression, and network analysis. First, descriptive statistics, partial correlation analysis (controlling for age, gender, educational level, participant group, and handedness), and linear regression analysis were conducted using SPSS 26.0 to examine the effects of spatial anxiety and wayfinding strategies on spatial navigation ability. Subsequently, network analysis was performed using R 4.5.1 to investigate the network structure among the dimensions and items of three spatial navigation-related scales: the SBSOD, the Spatial Anxiety Scale, and the Wayfinding Strategy Scale. Unlike Structural Equation Modeling (SEM), which tests predefined causal paths, network analysis estimates conditional dependencies between variables in a data-driven manner without assuming a causal direction, making it more exploratory. This analysis adhered to the standardized guidelines outlined by Epskamp et al. (2018), which encompass five key steps: network estimation, network visualization, centrality estimation, network comparison, and the evaluation of network accuracy and stability. Regarding the demographic variables, gender, age, education level, and handedness were incorporated as additional nodes in the Gaussian Graphical Model alongside the questionnaire items. It should be emphasized that including demographic variables as nodes does not constitute statistical control for confounding effects. Given the near-complete separation of sex by group (with the pilot group being 99.1% male) and the substantial age difference between groups, the current data do not permit aviation expertise to be fully disentangled from demographic composition.

#### 2.3.1 Network construction

The network was constructed with 32 nodes, consisting of 28 items from the three questionnaires and 4 demographic variables (gender, age, education level, and handedness). A Gaussian Graphical Model (GGM) was employed, with edge weights estimated using the EBICglasso method, where edges represent regularized partial correlations between items. No absolute weight threshold was set for truncation, and for network visualization, the maximum edge width was set to the strongest edge within each group. Item-level data were used rather than total or dimension scores because item-level networks can reveal the fine-grained structure within dimensions, complementing traditional total score or dimensional correlation analyses. Estimation was performed using the R packages *qgraph* and *bootnet*.

#### 2.3.2 Network estimation

In accordance with the standardized guidelines for network analysis (Epskamp et al., 2018), regularized partial correlation networks were estimated for the total sample as well as for the subgroups (non-pilot control group and commercial airline pilots) using the qgraph package in R. In these networks, circular nodes represent the dimensions or items of the scales, and the connecting lines between nodes, termed edges, represent partial correlations. The thickness of an edge corresponds to the magnitude of the absolute partial correlation coefficient.

The estimation procedure for each network consisted of two key steps. First, a GGM was employed to estimate pairwise association parameters among all nodes. Second, to mitigate the risk of identifying spurious associations, the Least Absolute Shrinkage and Selection Operator (LASSO) regularization technique was applied (Epskamp et al., 2018). This method shrinks smaller, likely negligible edge weights to zero, thereby promoting a more parsimonious and interpretable network structure and enhancing the precision in identifying the underlying network topology (van Borkulo et al., 2014). Specifically, the tuning hyperparameter gamma in the EBICglasso estimation was set to 0.5.

#### 2.3.3 Network visualization

For the total sample, as well as for the non-pilot control group and commercial airline pilot subgroups, network visualizations were generated using the *Fruchterman-Reingold* algorithm for node layout. In these visualizations, positive and negative edges are depicted in blue and red, respectively, with edge thickness corresponding to the absolute strength of the partial correlation between connected nodes. Nodes positioned in closer proximity within the visualization indicate stronger and/or more numerous connections between them. To enable direct visual comparison across the three networks (specifically, the item networks constructed from the SBSOD, the Spatial Anxiety Scale, and the Wayfinding Strategy Scale, respectively), the *averageLayout* function from the *qgraph* R package was employed. This function computes a unified node layout based on the average spatial positions of each node across all specified networks, thereby ensuring that identical nodes maintain consistent relative positions across each plotted network.

#### 2.3.4 Centrality estimation

To identify the most influential nodes within the network, centrality analysis was conducted. Node centrality refers to the structural importance of a node within the network’s topology. Several centrality indices, including Strength, Closeness, Betweenness, and Expected Influence (EI), were computed using the *centralityPlot* function from the *qgraph* package in R. The results were visualized using standardized *z*-scores and ordered by the Strength centrality metric for comparative clarity (Robinaugh et al., 2016).

#### 2.3.5 Network comparison test

A Network Comparison Test was performed to statistically compare the network structures of the non-pilot control group and commercial airline pilots. This permutation-based analysis assessed the invariance of four key network properties, namely overall network structure, global strength, specific edge weights, and node centrality indices. A total of 1,000 permutation iterations were executed to generate the null distribution for these comparisons. Statistical significance was determined at *α* = 0.05 for all omnibus tests (van Borkulo et al., 2022). To control the family-wise error rate resulting from multiple comparisons of individual edges and centrality indices, the *Holm-Bonferroni* correction procedure was applied. This analytical approach aimed to identify group-specific core features or critical pathways within the psychological networks.

#### 2.3.6 Accuracy and stability estimation

The accuracy and stability of the estimated network were assessed using the *bootnet* package in R. Edge weight accuracy was quantified by computing 95% confidence intervals via nonparametric bootstrapping (with 1,000 bootstrap samples), with narrower intervals indicating greater estimation precision. Network stability was evaluated using a case-dropping subset bootstrap procedure. This method involves iteratively removing random subsets of participants of increasing size, re-estimating node centrality at each step, and calculating the correlation between the centrality indices derived from these reduced samples and those from the full sample. The correlation stability coefficient (CS-coefficient) was then computed, defined as the maximum proportion of cases that can be dropped while maintaining, with 95% probability, a correlation of at least 0.7 between the original and subset-based centrality indices. In accordance with established methodological guidelines, a CS-coefficient above 0.25 is considered indicative of acceptable stability, while a value exceeding 0.5 denotes good stability (Epskamp et al., 2018).

## 3 Results

### 3.1 Descriptive statistics and correlations

**Figure 1:**
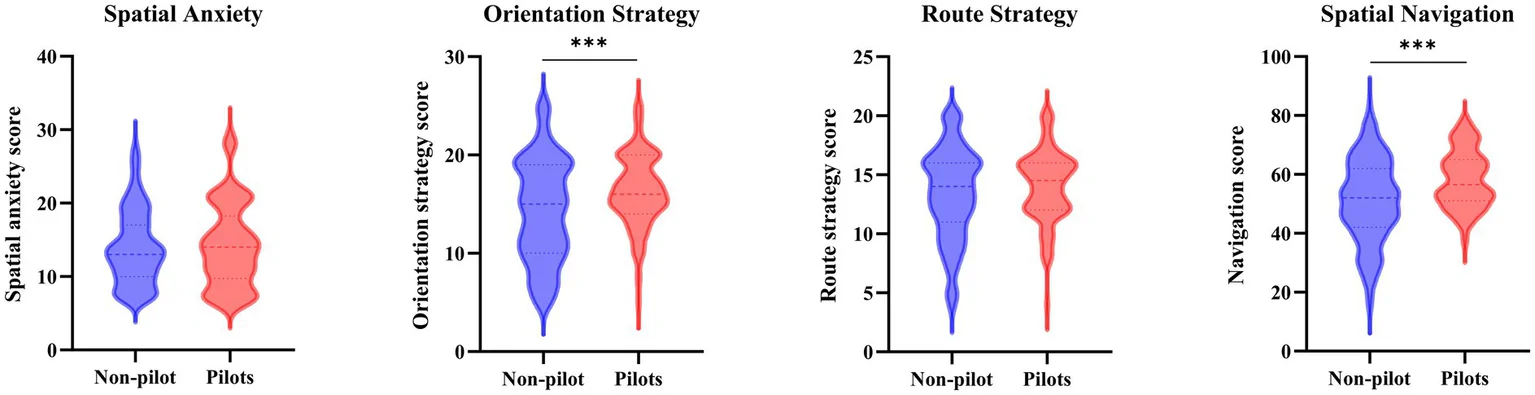
Differences in spatial anxiety, navigation strategy use (orientation/route), and Santa Barbara Sense of Direction Scale score between non-pilot control group and commercial airline pilot. “***”: *p* < 0.001.

Group comparisons between Civilian airline pilots and the non-pilot control group were conducted across four key metrics including spatial anxiety, route strategy, orientation strategy, and SBSOD scores. Nonparametric Mann–Whitney U tests were employed for these comparisons as Shapiro–Wilk tests indicated violations of the normality assumption for several variables (*p* < 0.05). The analysis revealed statistically significant differences for both orientation strategy (*Z* = −3.503, *p* < 0.001) and SBSOD scores (*Z* = −4.426, *p* < 0.001), with pilots achieving higher scores. Conversely, differences for spatial anxiety (*Z* = 0.110, *p* = 0.912) and route strategy (*Z* = −1.224, *p* = 0.221) were not statistically significant. These results are visually summarized in Figure 1.

To statistically control for the covariates of group identity, age, biological sex, dominant hand, and educational level, partial correlation analyses were performed. The results indicated a significant negative correlation between spatial anxiety and SBSOD scores (*r* = −0.319, *p* < 0.001). Conversely, both orientation strategy (*r* = 0.590, *p* < 0.001) and route strategy (*r* = 0.449, *p* < 0.001) exhibited significant positive correlations with SBSOD scores.

### 3.2 Linear regression analysis

**Figure 2:**
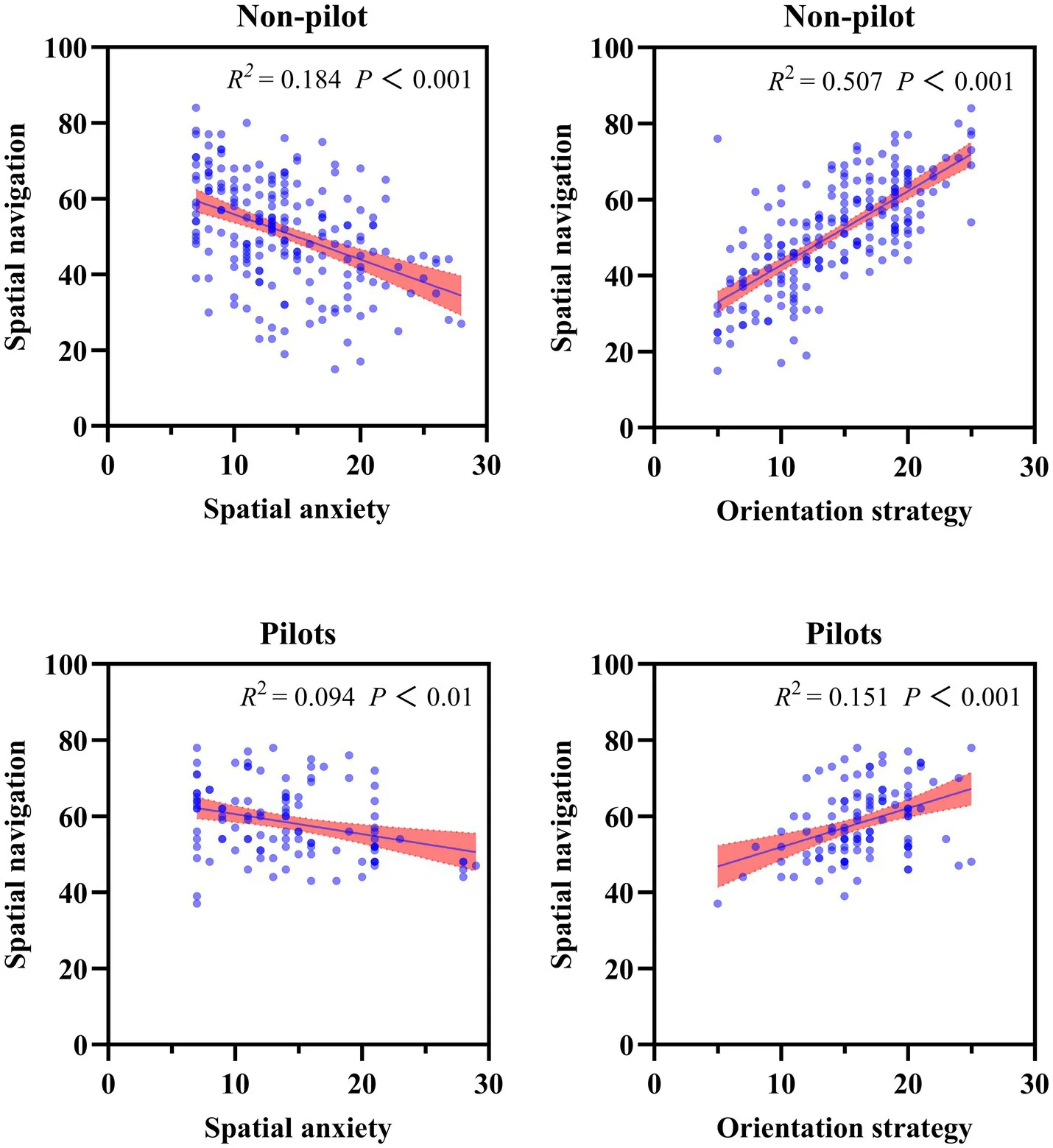
Relationships between spatial anxiety, orientation strategy, and Santa Barbara Sense of Direction Scale Score in non-pilot control group and commercial airline pilot.

Separate multiple linear regression analyses were conducted for the non-pilot control group and airline pilots to examine the distinct contributions of spatial anxiety, orientation strategy, and route strategy to SBSOD scores, while controlling for gender, age, dominant hand, and educational level. For the non-pilot control group, spatial anxiety emerged as a significant negative predictor (*β* = −0.185, SE = 0.15, *t* = −3.45, *p* = 0.001) and orientation strategy as a significant positive predictor (*β* = 0.517, SE = 0.202, *t* = 6.990, *p* < 0.001); route strategy was not a significant predictor. A highly convergent pattern was observed for pilots, with spatial anxiety (*β* = −0.289, SE = 0.151, *t* = − 3.276, *p* = 0.001) and orientation strategy (*β* = 0.324, SE = 0.302, *t* = 2.823, *p* = 0.006) again constituting significant negative and positive predictors, respectively, while route strategy again failed to reach significance (see Figure 2).

### 3.3 Network comparison between university students and pilots

#### 3.3.1 Network

**Figure 3:**
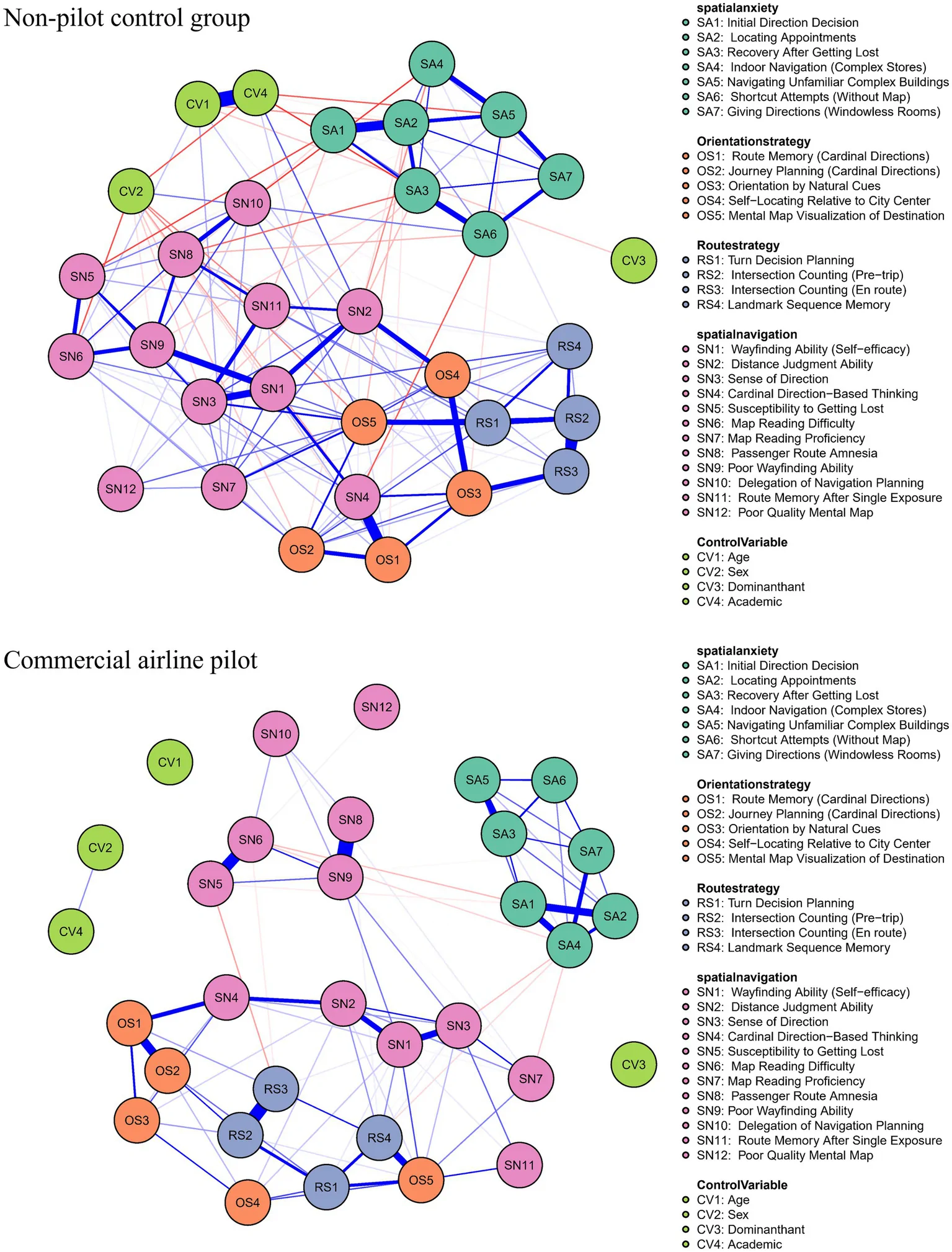
Network structure visualizations for non-pilot control group and commercial airline pilot. Blue lines represent positive associations, red lines negative ones; The thickness and saturation of a line correspond to its association strength.

For the non-pilot control group, a regularized partial correlation network comprising 32 nodes and 496 possible edges was estimated, of which 155 edges had non-zero weights (Figure 3). The edge with the strongest weight was between nodes RS3 and RS2 (*r* = 0.416), followed by the edge between OS1 and SN4 (*r* = 0.413). For the airline pilot group, a comparable network structure with an identical number of nodes and possible edges (32 and 496, respectively) was constructed, yielding 87 non-zero edges (Figure 3). Within this network, the strongest edge was found between nodes SN9 and SN8 (*r* = 0.425), and the second strongest edge was between nodes RS3 and RS2 (*r* = 0.391). For visualization purposes, edge thickness represents the strength of the regularized partial correlation. The maximum edge width in each network was set to the strongest edge within that network; no absolute cutoff was applied, so all non-zero edges are displayed.

#### 3.3.2 Centrality

**Figure 4:**
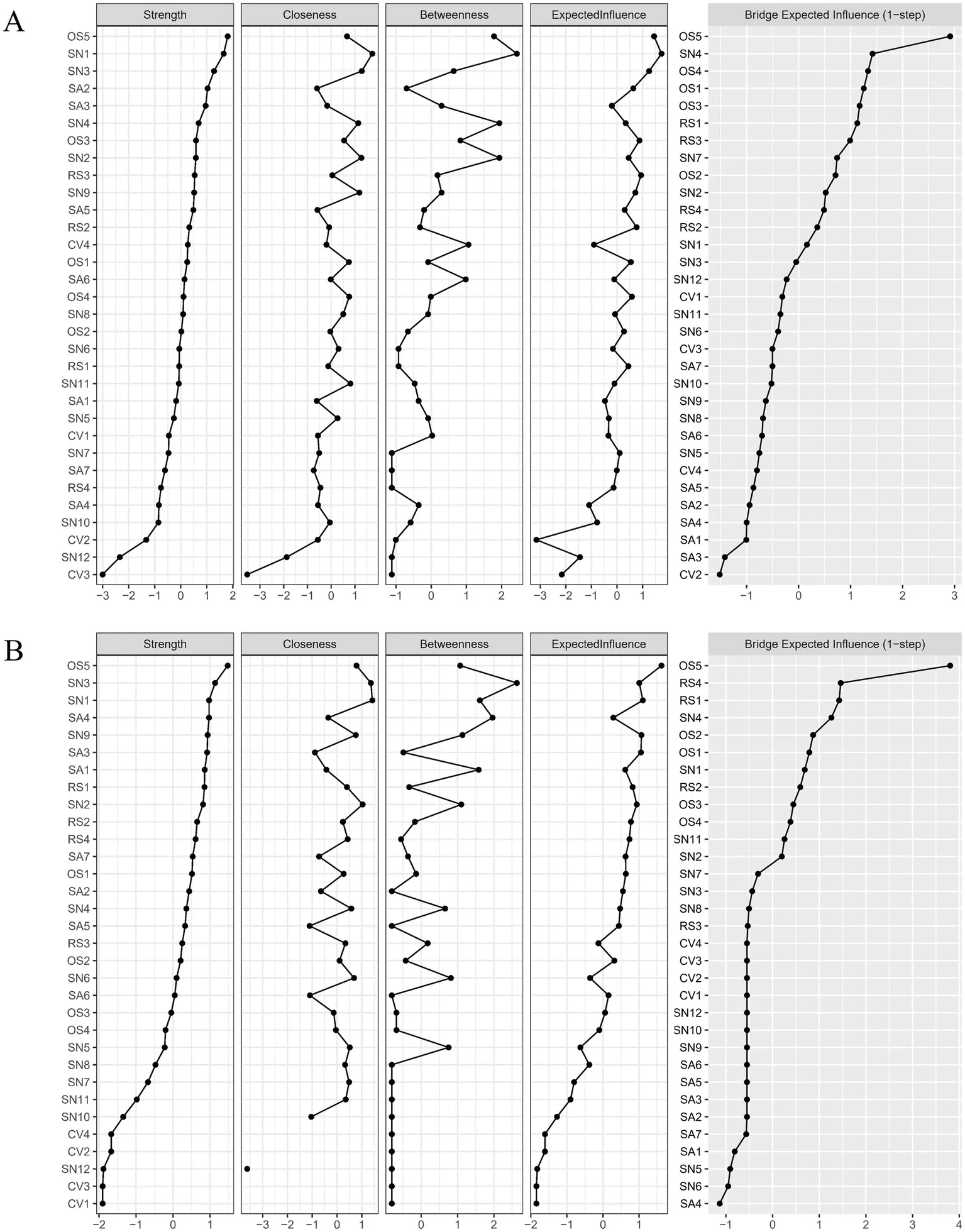
Non-standardized estimates of expected influence and bridge expected influence for university students and airline pilots. **(A)** Non-pilot control group; **(B)** Commercial airline pilot.

To identify core nodes in the networks, the Expected Influence (EI) of each node was calculated. The three nodes with the highest expected influence in the non-pilot control group were SN1, OS5, and SN3, and in the pilot group the top three were OS5, SN3, and SN1, indicating that both groups shared several central nodes, though the relative prominence of OS5 was greater in pilots (Figure 4). Additionally, Bridge Expected Influence (BEI) was examined, which indicated that node OS5 functioned as the most central bridge connecting distinct dimensions between the student and pilot networks.

#### 3.3.3 Network accuracy and stability

**Figure 5:**
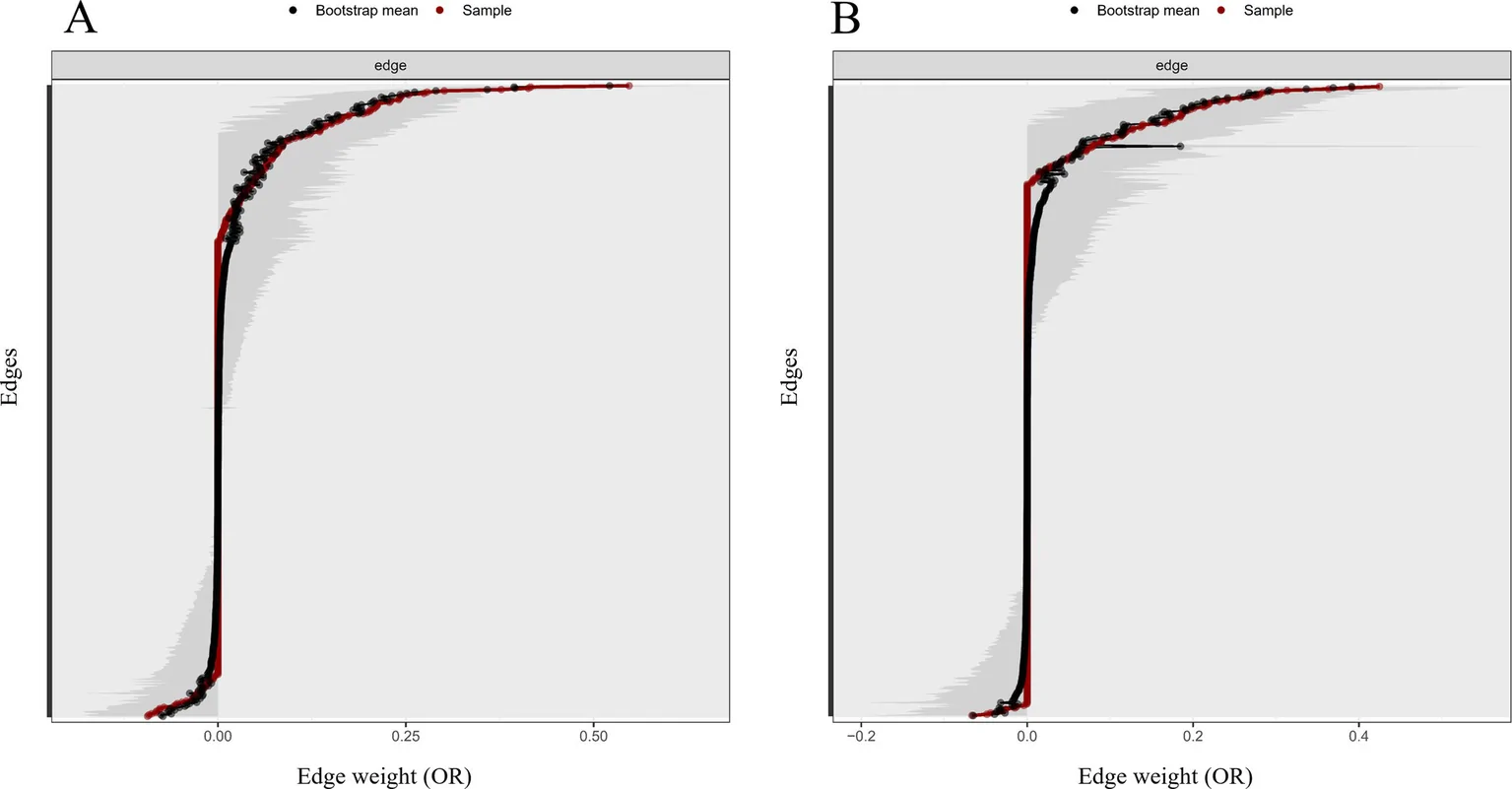
Bootstrapped confidence intervals of the edge weights for the non-pilot control group **(A)** and the commercial airline pilot network **(B)**.

The accuracy and stability of the estimated networks were assessed. Bootstrap analysis of edge weights indicated good estimation precision for both the non-pilot control group and airline pilot networks. As shown in Figure 5, the 95% bootstrap confidence intervals for the vast majority of edges exhibited substantial overlap, suggesting limited sampling variability.

**Figure 6:**
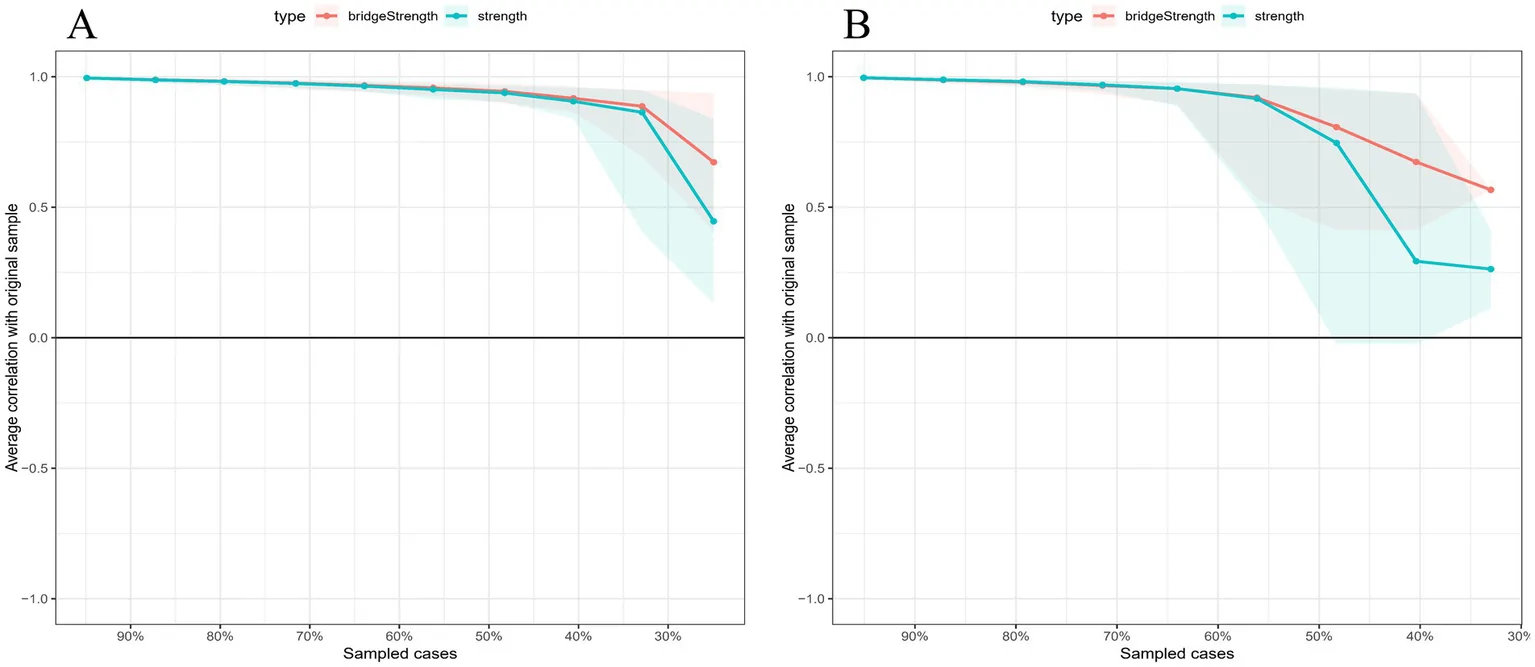
Stability of centrality indices under case-dropping subset bootstrap for the non-pilot control group **(A)** and the commercial airline pilot network **(B)**.

Stability analysis, quantified via the correlation stability coefficient (CS-coefficient), revealed that neither network reached the ideal threshold (CS > 0.5) for node strength centrality. The correlation stability (CS) coefficient for strength centrality was 0.44 for the non-pilot control network and 0.28 for the pilot network (Figure 6). The CS coefficient for the pilot group was close to the minimum recommended threshold of 0.25, suggesting that the strength centrality estimates for this group should be interpreted with caution (Epskamp et al., 2018).

#### 3.3.4 Network comparison

To formally compare the network structures between university students and airline pilots, the Network Comparison Test (NCT) was conducted. The results revealed a statistically significant difference in the invariance of network structure between the two groups (*p* < 0.001). In contrast, no significant difference was found in the invariance of global strength (*p* = 0.376). Subsequent comparisons of individual edge weights and node centrality indices were performed using Holm-Bonferroni correction. No single edge exceeded the corrected significance threshold (all adjusted *p* > 0.05), suggesting that the structural difference was distributed across the network rather than localized to a specific subset of connections. Centrality invariance tests further indicated that neither strength nor expected influence differed significantly between groups for any node (all adjusted *p* > 0.05). The expected influence of item OS5, which ranked among the most central nodes in both groups, did not approach significance (*p* = 0.997). Comprehensive node-level centrality invariance test statistics and Holm-adjusted *p*-values for all nodes are presented in Table 2. While the overall architecture of inter-item dependencies differed, global connectivity appeared comparable, and the observed reconfiguration was not driven by any isolated edge or node.

**Table 2:** Node centrality invariance test statistics and Holm-adjusted *p*-values.

Node	*C*_strength	*p*	*C*_expectedInfluence	*p*
SA1	−0.148	0.899	−0.294	0.855
SA2	0.325	0.855	0.090	0.915
SA3	0.138	0.909	−0.350	0.855
SA4	−0.368	0.855	−0.387	0.855
SA5	0.214	0.880	0.018	0.967
SA6	0.216	0.880	−0.021	0.967
SA7	−0.153	0.899	−0.145	0.899
OS1	0.085	0.945	0.033	0.967
OS2	0.130	0.899	0.051	0.957
OS3	0.368	0.855	0.335	0.855
OS4	0.291	0.855	0.291	0.855
OS5	0.179	0.880	0.001	0.997
RS1	−0.114	0.899	−0.062	0.945
RS2	0.059	0.945	0.059	0.945
RS3	0.254	0.855	0.417	0.855
RS4	−0.222	0.855	−0.222	0.855
SN1	0.309	0.855	0.268	0.855
SN2	0.074	0.945	−0.097	0.916
SN3	0.153	0.899	0.142	0.899
SN4	0.257	0.868	0.019	0.967
SN5	0.198	0.899	0.170	0.899
SN6	0.145	0.915	0.134	0.899
SN7	0.297	0.880	0.367	0.855
SN8	0.380	0.855	0.168	0.899
SN9	0.013	0.977	−0.054	0.957
SN10	0.420	0.855	0.236	0.880
SN11	0.508	0.855	0.333	0.855
SN12	0.198	0.855	0.198	0.855
CV1	0.719	0.855	0.571	0.096
CV2	0.403	0.880	−0.432	0.855
CV3	0.027	0.967	−0.027	0.957
CV4	0.837	0.064	0.307	0.855

## 4 Discussion

A psychological network analysis was conducted to compare the self-reported psychological network architecture of spatial strategies between the pilot group and the non-pilot control group. At the measure level, the pilot group reported higher scores only in orientation strategy and self-reported sense of direction scores, with no significant group differences observed for spatial anxiety or route strategy. Network-level comparisons revealed a statistically significant difference in the global network structure between the two groups, whereas global connectivity strength appeared comparable. However, following Holm-Bonferroni correction, no individual edges or nodes differed significantly between the two groups, suggesting that the structural difference was distributed across the network rather than being localized to specific connections or central nodes. These findings indicate that the observed reconfiguration in inter-item dependencies was not driven by any isolated edge or node, and the results should be interpreted strictly within the context of self-reported questionnaire data. No causal inferences regarding cognitive mechanisms or physiological substrates can be drawn from the present network data.

### 4.1 Structural differences in self-reported spatial strategy networks

The observed pattern of structural reorganization with comparable global connectivity is consistent with descriptions of certain economy-related hypotheses, although the efficiency implications of these patterns cannot be directly inferred from the correlational network data. This finding aligns with the neural efficiency hypothesis in cognitive neuroscience, which posits that expert performance arises from more streamlined, task-specific neural activity rather than a global augmentation of cognitive resources (Neubauer and Fink, 2009; Malanchini et al., 2020). Extending this perspective to the level of self-reported psychological networks, although the pilot group did not exhibit stronger global network connectivity than the non-pilot control group, the inter-item dependencies underwent reorganization. This suggests that the self-reported psychological network architecture of the pilot group may be characterized by more differentiated inter-item relationships rather than an overall increase in connectivity strength.

A detailed analysis of centrality metrics indicates that the two groups’ networks showed substantial overlap in core nodes such as OS5, SN1, and SN3, with differences primarily reflected in the global configuration of edge weights rather than the prominence of individual nodes. It is important to note that following Holm-Bonferroni correction, no individual edges or nodes differed significantly between the two groups (including OS5, expected influence *p* = 0.997). The variation in OS5 centrality was merely a slight adjustment in descriptive ranking and lacks statistical inferential support. The non-pilot control group exhibited a tendency for specific strategy clusters to aggregate around the node representing generalized self-efficacy (SN1). This may suggest that, in the absence of fully developed professional skills, individuals rely on broad confidence beliefs as a self-reported organizational strategy. In contrast, the pilot group’s network appeared to show a relative clustering around the capacity for cognitive map construction (OS5). This descriptive pattern suggests that prolonged professional training may be associated with a shift in reported cognitive function from a generalized motivational level toward concrete, task-relevant cognitive operations (Chersi and Burgess, 2015; Mouratille et al., 2022). However, the current cross-sectional design cannot determine whether these network differences reflect the effects of flight training or occupational self-selection, as individuals with a stronger propensity for map-based strategies may be more likely to become pilots.

Regarding the structural position of specific items, while OS5 was an important node in both networks, its centrality in the pilot group did not represent a statistically significant elevation but rather a descriptive variation. Thus, it should not be interpreted as a transformation from a discrete skill to an organizing principle for the entire spatial cognitive system. These descriptive findings may complement prior conclusions regarding pilots’ reliance on spatial strategies by highlighting the relative positions of items within the self-reported psychological network architecture, although caution is warranted given the lack of statistical significance (Sutton et al., 2014; Giancola et al., 2021; Sungho et al., 2025).

Additionally, Bridge centrality analysis suggested that OS5 in the pilot group’s network appeared to connect disparate psychological dimensions, including strategy application, self-assessment, and emotional response. This descriptive role is consistent with perspectives that emphasize functional differentiation coupled with enhanced integration, whereby self-reported networks may exhibit specialized sub-functions but also exhibit more efficient coordination among them (Alperson-Afil et al., 2020).

The network analytic findings partially converge with those derived from traditional regression analysis. Linear regression identified orientation strategy as a common key predictor of navigation performance for both groups. Network analysis further indicated that the structural position and connective role of this factor appeared to differ between the two self-reported psychological networks. This contrast underscores the unique value of psychological network analysis: it moves beyond identifying which factors are important to delineating how these factors interact and organize within a dynamic self-reported psychological network architecture. This structural insight provides a descriptive framework for understanding the inter-item relationships in self-reported spatial strategies.

### 4.2 Implications for selection and training

The current findings may offer theoretical considerations and practical implications for selection and training within aviation human factors. The results indicate that differences between the pilot group and the non-pilot control group in spatial cognition extend beyond disparities in isolated abilities to variations in the organizational structure of the underlying self-reported psychological network architecture. Traditional selection and training paradigms may benefit from considering the functional configuration of entire networks, although it is important to note that training may be associated with these network characteristics, but the causal direction requires confirmation through longitudinal studies.

Regarding selection, current approaches predominantly rely on tests of isolated cognitive abilities, such as mental rotation and spatial relation judgment (Causse et al., 2011). While these assessments effectively identify individuals with high general spatial ability, they may fail to capture the coordinated organization among self-reported cognitive components. A key insight from this study arises from the divergence between regression and network analysis results: although orientation strategy was a common predictor of navigation performance across both groups, the network hub underpinning this strategy and the overall structural configuration appeared to differ. This suggests that assessing a candidate’s spontaneous tendency to construct cognitive maps and their reliance on such integrative processes may possess potential predictive value than measuring isolated spatial abilities alone. Future selection instruments could therefore incorporate evaluations of allocentric reasoning and the habitual use of survey-level representations. For example, scenario-based simulation tasks could be designed to gage a candidate’s propensity to spontaneously form and utilize mental representations of complex environments. This human-factors-oriented innovation in selection methodology aims to identify individuals who either innately possess or demonstrate a high potential for developing distinct self-reported psychological network configurations.

At the training level, the findings suggest that training objectives should extend beyond enhancing foundational procedural knowledge, such as route learning, to deliberately foster the development of expert-level situational awareness. This involves cultivating the ability to construct, maintain, and dynamically update survey-level mental representations, specifically cognitive maps. Simulator-based training curricula could be designed for this purpose (Do et al., 2021). For instance, trainees might be instructed to actively generate and verbally report their mental maps in specific scenarios—such as complex airspace or unfamiliar airport environments—and to update these representations dynamically in response to simulated changes in weather or traffic, while temporarily reducing their reliance on external cues like primary flight displays. This “deliberate practice in cognitive mapping” aims to directly reinforce the functional centrality of cognitive map construction within the self-reported network. It is important to note that training may be associated with these self-reported network characteristics, but the causal direction awaits confirmation from longitudinal studies (Madl et al., 2015). Moreover, given the potential constraining role of spatial anxiety within the observed networks, targeted interventions for anxiety management, including cognitive reappraisal and stress exposure training, could not only reduce subjective anxiety but also relate to the overall resilience and functional integration of the self-reported psychological network (Nori et al., 2023).

A balanced perspective is needed regarding the interaction between innate predisposition and training-induced plasticity. Longitudinal studies indicate that novice pilots’ spatial abilities can improve with accumulated flight experience, suggesting an association with training (van Weelden et al., 2022). Simultaneously, the network characteristics observed in pilot group likely stem partly from long-term self-selection and the reinforcement of specific cognitive styles within the occupational environment (Minoretti et al., 2025). Therefore, the practical implications involve placing considerable emphasis on systematic, explicit training, while acknowledging that training may be associated with these network features, but the causal direction awaits confirmation from longitudinal studies. Assessment tools based on network psychometrics may in the future provide deeper insights into a candidate’s self-reported psychological network architecture, extending beyond what traditional tests can measure. This approach may lay a foundation for developing integrated, personalized systems for cognitive capacity development in aviation personnel that bridge selection and training.

### 4.3 Limitations and future directions

Several limitations of the present study warrant candid discussion. The cross-sectional design precludes causal inferences regarding the observed network differences. It remains unclear whether these differences stem from adaptive reorganization due to prolonged professional training or from pre-existing factors related to self-selection during pilot recruitment (Sutton et al., 2014), This cross-sectional design further precludes any inference regarding the causal direction between training exposure and network reconfiguration; whether flight training contributes to network changes or whether individuals with particular network profiles self-select into aviation careers cannot be determined from the current data. Additionally, the pilot group was 99.1% male and approximately 11 years older than the non-pilot control group. Although gender, age, education level, and handedness were incorporated as additional nodes in the network model, this approach does not constitute statistical control for demographic confounding effects. Given the near-complete separation of sex by group, the present data do not permit aviation expertise to be fully disentangled from demographic composition. These demographic disparities represent potential confounding variables; group differences in network structure cannot be attributed solely to flight expertise, as gender and age may partially account for the observed network variations.

All data were derived from self-report questionnaires, which capture subjective strategy preferences rather than objective cognitive processes. The resulting network structures thus reflect self-reported psychological characteristics and should not be equated with objective navigational competence or underlying neurocognitive architectures. While these measures effectively capture subjective cognitive characteristics, they may not fully correspond to objective performance in actual navigation tasks.

Regarding the stability of network centrality metrics, the correlation stability (CS) coefficient for node strength centrality in the pilot network was 0.28. Although this meets the minimum acceptable threshold (>0.25), it falls short of the ideal level (0.5), indicating that while the ranking of core nodes is interpretable, the precision of their estimated values should be interpreted with caution (Epskamp et al., 2018). The CS coefficient for strength centrality in the non-pilot control group was 0.44, surpassing the recommended minimum threshold of 0.25 and suggesting acceptable stability for that network. However, neither group met the ideal threshold of 0.50, and centrality comparisons should therefore be interpreted with due caution. Furthermore, the Chinese version of the spatial strategy questionnaire used in this study has undergone formal translation and validation procedures in Chinese populations, with its reliability and validity supported. This established psychometric evidence mitigates concerns regarding measurement invariance across groups.

Several directions for future research emerge from these limitations. Longitudinal studies tracking flight trainees from novice to proficient stages are needed to empirically examine the relationship between training interventions and network restructuring and to clarify the interaction between innate predisposition and experience-dependent plasticity. Replicating the current network models in larger, independent samples is essential to enhance the stability of centrality estimates and verify their generalizability. Future work could combine psychometric network modeling with objective behavioral measures, such as virtual reality navigation and direction estimation tasks, to enable multi-level validation (Hevey, 2018; Malanchini et al., 2020; Jing et al., 2023). Future research may also consider including nutritional biomarkers and other physiological indicators to examine potential associations, although this direction remains purely speculative and beyond the scope of the current data.

## 5 Conclusion

The present study compared the self-reported spatial strategy networks of the pilot group and the non-pilot control group, revealing significant differences in global network structure alongside comparable global strength. Following Holm-Bonferroni correction, no individual edges or centrality indices reached statistical significance, suggesting that these differences reflect distributed micro-adjustments in edge configuration rather than isolated nodal shifts. Core nodes assessing cognitive map preference and sense of direction occupied high-centrality positions in both groups, though rank-order differences did not reach statistical significance. The cross-sectional design precludes causal inferences, indicating that these network variations may reflect training effects, occupational self-selection, or a combination of both. In addition, the pronounced differences in sex and age distributions between the two groups may also have contributed to the observed network patterns, and the current data do not permit these demographic factors to be disentangled from the effects of professional expertise. Furthermore, as all data were derived from self-report questionnaires, these network architectures represent subjective strategy preferences rather than objective navigational competence or neurocognitive structures.

## Data Availability

The raw data supporting the conclusions of this article will be made available by the authors, without undue reservation.
